# Integration-deficient lentivectors: an effective strategy to purify and differentiate human embryonic stem cell-derived hepatic progenitors

**DOI:** 10.1186/1741-7007-11-86

**Published:** 2013-07-19

**Authors:** Guanghua Yang, Karim Si-Tayeb, Sébastien Corbineau, Rémi Vernet, Régis Gayon, Noushin Dianat, Clémence Martinet, Denis Clay, Sylvie Goulinet-Mainot, Gérard Tachdjian, Gérard Tachdjian, Deborah Burks, Ludovic Vallier, Pascale Bouillé, Anne Dubart-Kupperschmitt, Anne Weber

**Affiliations:** 1 INSERM U 972, IFR 93, Bicêtre Hospital, and Paul Brousse Hospital, Villejuif F-94807, France; 2 Université Paris-Sud, UMR-S972, IFR 93, Bicêtre Hospital and Paul Brousse Hospital, Villejuif F-94807, France; 3 Vectalys SAS, Canal Biotech 2, 3 rue des satellites, Toulouse F-31400, France; 4 CIBER de Diabetes y Obesidad, Centro de Investigación Principe Felipe, Eduardo Primo Yúfera 3, Valencia 46012, Spain; 5 Department of Cytogenetics, Béclère Hospital, Clamart F-92141, France; 6 Department of Surgery, The Anne McLaren Laboratory for Regenerative Medicine, Cambridge CB2 0SZ, UK

**Keywords:** Tissue-specific progenitor cells, Hepatoblasts, Purification, Non-integrating lentiviral vectors

## Abstract

**Background:**

Human pluripotent stem cells (hPSCs) hold great promise for applications in regenerative medicine. However, the safety of cell therapy using differentiated hPSC derivatives must be improved through methods that will permit the transplantation of homogenous populations of a specific cell type. To date, purification of progenitors and mature cells generated from either embryonic or induced pluripotent stem cells remains challenging with use of conventional methods.

**Results:**

We used lentivectors encoding green fluorescent protein (GFP) driven by the liver-specific apoliprotein A-II (APOA-II) promoter to purify human hepatic progenitors. We evaluated both integrating and integration-defective lentivectors in combination with an HIV integrase inhibitor. A human embryonic stem cell line was differentiated into hepatic progenitors using a chemically defined protocol. Subsequently, cells were transduced and sorted at day 16 of differentiation to obtain a cell population enriched in hepatic progenitor cells. After sorting, more than 99% of these APOA-II-GFP-positive cells expressed hepatoblast markers such as α-fetoprotein and cytokeratin 19. When further cultured for 16 days, these cells underwent differentiation into more mature cells and exhibited hepatocyte properties such as albumin secretion. Moreover, they were devoid of vector DNA integration.

**Conclusions:**

We have developed an effective strategy to purify human hepatic cells from cultures of differentiating hPSCs, producing a novel tool that could be used not only for cell therapy but also for *in vitro* applications such as drug screening. The present strategy should also be suitable for the purification of a broad range of cell types derived from either pluripotent or adult stem cells.

## Background

Cell therapy is now a viable alternative to liver transplantation for the treatment of chronic liver diseases. However, this approach is limited by the shortage of reproducible sources of human hepatocytes [1]. Human pluripotent stem cells (hPSCs) are an ideal source for generating hepatocytes because they can be isolated, expanded as clonal populations to generate sufficient numbers, and differentiated *in vitro.* Human embryonic stem cells (hESCs) remain the most reliable option, as they display an unlimited capacity for self-renewal.

We and others have generated hepatocyte-like cells from hESCs in animal-free conditions by recapitulating liver developmental stages [2-7]. However, although these differentiation protocols are relatively efficient, the presence of cells of an undesirable phenotype might pose health risks in the context of cell transplantation. Hence, for clinical applications, it is essential to transplant homogenous cell preparations that are highly enriched in the cells of interest, using a simple and reproducible procedure. Purified epithelial cell adhesion molecule EpCAM-positive cells from fetal and postnatal livers have been used to generate mature hepatocytes [8], but this marker is also expressed in the visceral endoderm and in several progenitor cell populations and cancers, and is associated with undifferentiated hESCs [9,10]. A cell surface marker specific to hepatic progenitors that could be used for the simple and efficient fluorescence-activated cell sorting (FACS) of hepatic progenitors differentiated from hESCs has not yet been identified.

Alternative approaches based on the use of conventional lentiviral vectors (lentivectors) are complicated by the problem of genomic integration of transgenes and viral DNA elements, potentially precluding their use for clinical applications. However, integrase-defective lentivectors (IDLVs) can be produced by introducing a mutation into the integrase gene, which specifically prevents lentivector DNA integration [11]. Transduction with IDLVs results in the generation of circular vector episomes, and the transgene is expressed from these non-integrated proviral forms, which are progressively lost in proliferating cells, resulting in transient gene expression. In a previous study, we designed a third-generation integrating lentivector (ILV) in which the gene encoding for green fluorescent protein (GFP) was under the control of the human liver-specific APOA-II promoter. We previously showed that this transgene is expressed in transduced primary simian hepatocytes both *in vitro* and *in vivo* after the transplantation of these transduced cells into animal models [12,13].

By combining 1) cell sorting using a hepatic-specific promoter, 2) high-titer preparations of purified ILVs and IDLVs, and 3) a specific integrase inhibitor, we created a robust and highly efficient method for purifying hESC-derived hepatic progenitors devoid of DNA integration.

## Results

### Hepatic specificity of reporter lentivector expression

**Figure 1:**
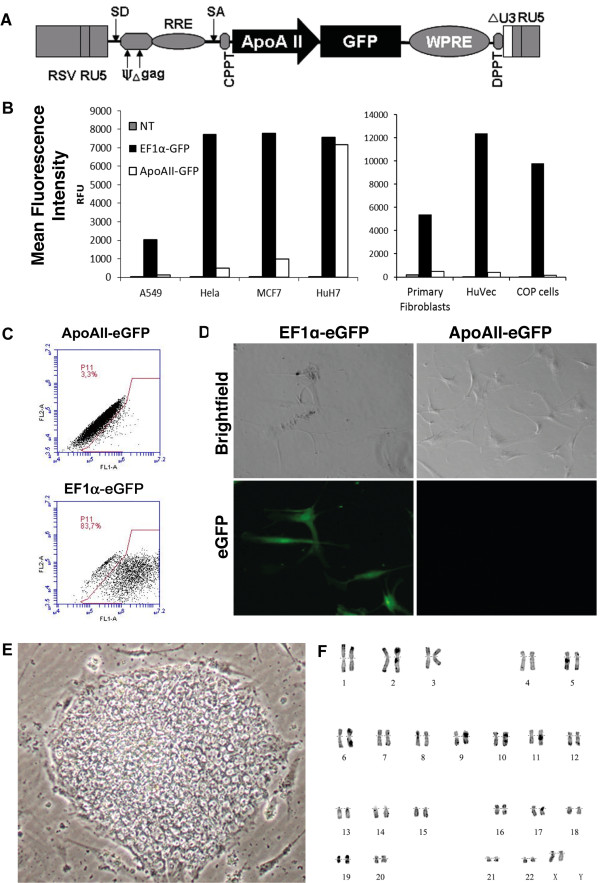
**Specificity of apolipoprotein A-II (APOA-II) promoter for hepatic cells. (A)** Schematic diagram of the APOA-II-green fluorescent protein (APOA-II-GFP) lentivector. **(B)** Relative mean fluorescence intensity (MFI) representing GFP expression was evaluated in various types of cells: different epithelial cell lines (A549, HeLa, MCF7), hepatoma cells (HuH7), primary fibroblasts, human umbilical vein endothelial cells (HUVECs), and human pancreatic (COP) cells. Non-transduced control (NT, grey bars), transduced with elongation factor (EF)1α lentivector (black bars), or with APO-AII-GFP (white bars) lentivector. **(C)** Fluorescence-activated cell sorting (FACS) analysis of GFP-expressing fibroblasts 3 days after transduction with APOA-II-enhanced (e)GFP or EF1α-eGFP lentivectors. **(D)** Phase-contrast and fluorescence micrographs after the transduction of mesenchymal stem cells (MSCs) with EF1α-GFP and APOA-II-GFP lentivectors. **(E)** Phase-contrast image of a representative H9 cell colony transduced with APOA-II-GFP lentivector. **(F)** Karyotype analysis of H9 cells transduced with APOA-II-GFP lentivector.

We first investigated the specificity of the APOA-II promoter by transducing various cell lines with APOA-II-GFP lentivector (Figure 1A). Whereas the ubiquitous elongation factor (EF)1α promoter was expressed in all cell lines tested, the APOA-II promoter induced high levels of GFP expression only in the hepatic cell line HuH7. GFP expression was not detected in the human epithelial cell lines tested (A549, Hela, MCF7) nor in the COP cell line derived from human pancreatic islet cells, which like hepatic cells, are of endoderm origin (Figure 1B). Because a meso-endoderm stage is common to both mesoderm and endoderm, we also verified the specificity of the APOA-II promoter in endothelial cells (human umbilical vein endothelial cells; HUVECs), primary human fibroblasts (Figure 1B), and primary mesenchymal stem cells (MSCs) (Figure 1D). Figure 1C shows a representative FACS analysis of primary fibroblasts transduced with either the elongation factor (EF)1α-GFP lentivirus or the APOA-II-GFP lentivirus.

Undifferentiated H9 cells transduced with APOA-II-GFP vectors at a multiplicity of infection (MOI) of 10 displayed normal hESC morphology (Figure 1E) and karyotype (Figure 1F) and, as expected, did not express GFP (not shown).

### Purification of hepatic progenitors by FACS

**Figure 2:**
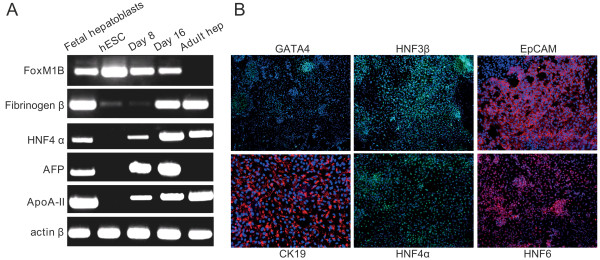
**Expression of specific markers by human embryonic stem cells (hESCs) differentiating to hepatic progenitors. (A)** Representative picture of four separate experiments showing transcript levels of hepatic cell markers on D8 and D16 of differentiation. **(B)** Representative immunofluorescence staining for human GATA binding protein (GATA)4, hepatic nuclear factor (HNF)3β, epithelial cell adhesion molecule (EpCAM), cytokeratin (CK)19, hepatic nuclear factor (HNF)4α , and HNF6.

**Figure 3:**
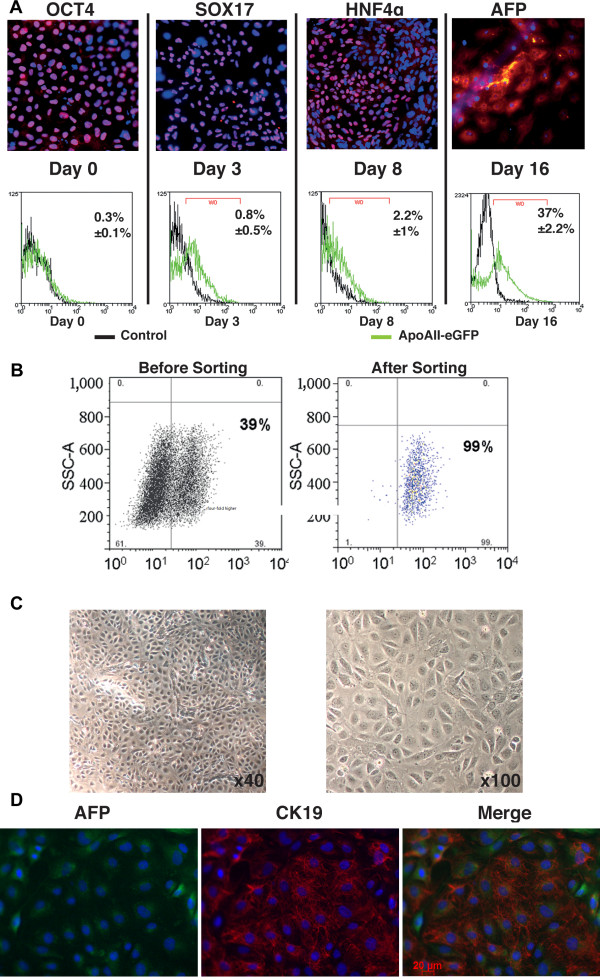
**Purification of hepatic progenitors upon differentiation of human embryonic stem cells (hESCs) transduced with apolipoprotein A-II-green fluorescent protein (APOA-II-GFP) lentivectors. (A)** Immunofluorescence staining showing expression of cell markers characteristic of hESCs (day 0; octamer-binding transcription factor (OCT)4), endoderm (day 3; sex determining region Y-box (SOX)17), hepatic endoderm (day 8; hepatic nuclear factor (HNF)4α), hepatic progenitors (day 16; α-fetoprotein (AFP) and representative fluorescence-activated cell sorting (FACS) analysis of the same cells showing time course of GFP expression after transduction of hESCs with APO-AII-GFP-integrating lentivector (ILV) (green lines) versus non-transduced cells (black lines). **(B)** Representative GFP fluorescence profile on day 16 of differentiation, before and after FACS. Left panel: GFP-positive cells of the low/right quadrant, representing 39% of the living cells, were sorted and subsequently analyzed, as illustrated in the right panel, where 99% were GFP-positive. **(C)** Phase-contrast images of sorted hESC-derived hepatic progenitors, 48 hours after plating. **(D)** Representative fields of immunofluorescence staining of sorted hepatic progenitor cells treated with α-fetoprotein (AFP) (green) and cytokeratin (CK)19 (red) antibodies.

To assess the suitability of the APOA-II reporter vector for a cell-sorting strategy, we directed the differentiation of transduced ES cells into hepatic progenitors [7], and the expression of APOA-II-GFP was investigated during the various stages of cell differentiation. As expected, hepatoblasts (day 16) expressed hepatic progenitor markers, such as α-fetoprotein (AFP), cytokeratin 19 (CK19), hepatic nuclear factor (HNF)4α and HNF6 (Figure 2A, B). From day 0 to day 8, early markers of differentiation, such as sex determining region Y-box (SOX)17 and HNF4α, were sequentially detected but less than 3% of the cells expressed GFP, reflecting the low level of APOA-II promoter activity at this stage (Figure 3A). On day 16 (hepatic progenitor stage), up to 39% of the cells expressed GFP (Figure 3A, B). Sorting at day 16 yielded a population highly enriched in ApoA-II-GFP-expressing cells (>99%) (Figure 3B). After sorting, GFP-positive cells were plated on type I collagen-coated plates and maintained in culture. After a 48-hour period, purified GFP-positive cells were homogeneously distributed, displayed the morphology of hepatic progenitors (Figure 3C), and expressed both CK19 and AFP (Figure 3D).

### Generation of a purified population of hepatic progenitors devoid of virus integration

Use of a conventional lentivector would result in major changes to the genetic material of the host genome, including potentially harmful mutations. Therefore, we aimed to develop a cell-purification strategy that would prevent permanent genome modification. EF1α-GFP and APOA-II-GFP were produced in an integration-defective form, with a GAG/POL packaging plasmid encoding the D64V mutant integrase [14,15].

We developed a protocol by transducing H9 cells with EF1α-GFP-IDLV at various time points (days 8 (not shown), 10 (not shown) and 13) of the differentiation protocol, and monitoring the kinetics of GFP expression from days 3 to 7 after transduction. The percentage of GFP-positive cells was highest on day 3 after transduction (see Additional file 1: Figure S1A), with 60% of the cells expressing GFP at an MOI of 30 when cells were transduced on day 13 of differentiation. Therefore, for subsequent experiments, we transduced hepatic cells on day 13 and sorted the fluorescent cell population 3 days later, that is, on day 16 of differentiation. Cells were transduced with purified EF1α-GFP-ILV and EF1α-GFP-IDLV to confirm the advantages of IDLV over ILV for sorting.

On day 3 after transduction, the proportions of GFP-IDLV cells and GFP-ILV cells were similar, owing to episome transcription (see Additional file 1: Figure S1B). However, the proportion of GFP-IDLV cells subsequently decreased to 1% 14 days after transduction, whereas the proportion of GFP-ILV-positive cells remained stable (approximately 50%) as expected (see Additional file 1: Figure S1B).

We also investigated the benefits of using raltegravir, an integrase inhibitor used in the clinical treatment of HIV infection, during the transduction protocol to prevent any residual vector integration [16]. Addition of raltegravir had no effect on the percentages of GFP cells on day 3 after transduction for either type of vector, owing to the presence of non-integrated forms, suggesting that this inhibitor has no effect on differentiation and/or transduction efficacy. On day 14 after transduction, the proportion of IDLV-transduced GFP-positive cells had decreased to less than 1%, in the presence or absence of raltegravir. By contrast, raltegravir decreased the fraction of GFP-ILV-positive cells to less than 1% (see Additional file 1: Figure S1B).

**Figure 4:**
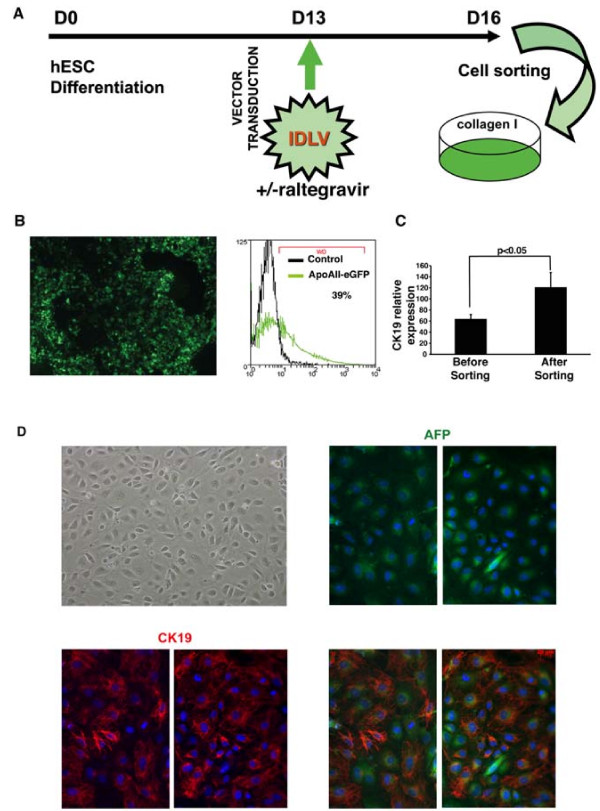
**Purification of hepatic progenitors after transduction with integrase-defective apolipoprotein A-II-green fluorescent protein (APOA-II-GFP) lentivectors. (A)** Flowchart summarizing protocol for purification of hepatic progenitor cells. **(B)** Representative GFP fluorescence analyses of the cell population carried out on at day 16 (3 days after transduction on day 13 of differentiation with the APOA-II-GFP integrase-defective lentivector (IDLV) by immunofluorescence microscopy (left panel) and fluorescence-activated cell sorting (FACS) analysis (right panel). **(C)** Cytokeratin (CK)19 transcript levels in hepatic progenitors were determined immediately before and after sorting (day 13 and 16 of differentiation, respectively) by quantitative reverse transcription (qRT)-PCR analysis. **(D)**. A phase-contrast image of human embryonic stem cells (hESC)-derived hepatic progenitors plated on type I collagen and cultured for 48 hours after sorting (magnification × 100). Immunofluorescence staining of representative fields of sorted hepatic cells treated with α-fetoprotein (AFP) (green) and CK19 (red) antibodies after 48 hours of culture on type 1 collagen.

The final protocol used for the purification of hepatic progenitors from hESCs is depicted in Figure 4A. As reported for APOA-II-GFP-ILV, about 39% of differentiated cells were positive for APOA-II-GFP-IDLV at the time of sorting (Figure 4B). After a 2-day culture period, quantitative reverse transcription-(RT)-PCR of GFP-sorted cells confirmed enrichment of CK19 expression compared with qRT-PCR performed on the day of sorting (Figure 4C). Several co-immunostaining experiments showed that these cells co-expressed CK19 and AFP, although at different levels, confirming their hepatic progenitor phenotype (Figure 4D).

### Differentiation of purified hepatic progenitors devoid of viral DNA integration

**Figure 5:**
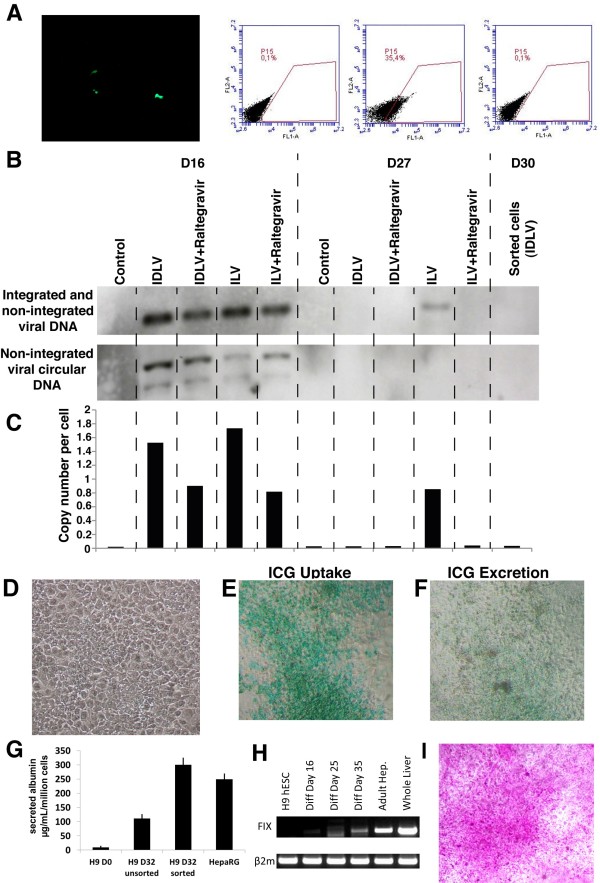
**Differentiation of hepatoblasts into hepatocyte-like cells free of vector integration. (A)** Fluorescence microscopy (left panel) 18 days after sorting and fluorescence-activated cell sorting (FACS) analysis of green fluorescent protein (GFP) expression from (left) non-transduced progenitors (left FACS panel), and transduced progenitors (middle) 3 days after transduction and (right) and 18 days after sorting. The numbers indicate the percentages of GFP-positive cells in the analyzed P15 gate. **(B)** Representative Southern blots of integrated and non-integrated forms (1-long terminal repeat (LTR) and 2-LTR circles) of the lentivector at 3 days and 14 days after transduction (days 16 and 27, respectively, of differentiation) in the presence or absence of raltegravir. **(C)** Total copy number of lentivectors was analyzed in the same cells as in **(B)** by quantitative-(q)PC). **(D)** Representative phase-contrast image of differentiating purified hepatic progenitors on day 14 after sorting. **(E**, **F)** Uptake and excretion of indocyanin green. **(G)** Albumin secretion of undifferentiated H9 cells, non-purified hepatic cells, purified hepatic cells and Hepa-RG hepatoma cell line in the culture medium was evaluated by ELISA. **(H)** Representative transcript levels of hepatocyte-specific clotting Factor IX (FIX) on days 16, 25, and 35 of differentiation. **(I)** Glycogen storage of the purified cells was assessed by periodic acid-schiff (PAS) staining, 14 days after sorting (day 30 of differentiation).

After sorting, cells were allowed to reach confluence in a serum-free medium previously defined for the culture of fetal hepatic progenitors, and then they were cultured in hepatocyte culture medium supplemented with hepatocyte growth factor (HGF) and Oncostatin M. On day 18 after sorting (day 34 of differentiation), we analyzed the GFP expression of the cells. Only a very small number of fluorescent cells were visible by fluorescence microscopy, and FACS analysis confirmed that no more than 0.1% of the cells were fluorescent, whereas at day 16 (3 days after transduction) up to 35% cells were transduced (Figure 5A).

Cells transduced with either ILV or IDLV in the presence or absence of raltegravir were analyzed or passaged on day 16 of differentiation (day 3 after transduction), and the presence of lentivector DNA forms were analyzed using the described probe (see Additional file 2: Figure S2). A band common to both integrated and two bands (ECOR1) specific for non integrated forms (one and two long terminal repeat (LTR) circle of the lentivector DNA were detected in all transduced cells (Figure 5B). At day 14 after transduction (day 27 of differentiation), lentivector DNA could be detected only in cells transduced with ILV in the absence of raltegravir (Figure 5B). Integrated viral DNA was absent from cells transduced either with IDLV or with IDLV or ILV in the presence of raltegravir. In purified cells at day 30 of differentiation (day 14 of culture), no integrated or episomal DNA derived from lentivectors was detected (Figure 5B). qPCR of genomic DNA confirmed the absence of viral DNA from all samples on day 27 of differentiation, with the exception of cells transduced with ILV in the absence of raltegravir (Figure 5C). The threshold of detection was analyzed by qPCR using a clonal cell line control (HCT116) which, after transduction with a GFP lentivirus, contained one lentiviral integration per cell (see Additional file 3: Figure S3). At a dilution of 1 in 2,000, integration of viral DNA was 10 times higher than the background in control non-transduced cells, and it was 7 to 7.5 time higher at dilutions of 1 in 4,000, 1 in 5,000, and 1 in 10,000. Thus, these studies established the limit of detection of an integration event of less than 1 in 10,000.

We then investigated whether hepatic progenitors were able to differentiate further into more mature hepatocytes. On day 16 after sorting (day 32 of differentiation), the human progenitor-derived hepatocytes had acquired a morphology resembling that of hepatocytes (Figure 5D), and had the functional characteristics of mature human hepatocytes. These cells could incorporate and export indocyanin green (ICG) (Figure 5E, F), secrete albumin (Figure 5G), express clotting Factor IX (FIX) mRNA (Figure 5H), and store glycogen (Figure 5I).

**Figure 6:**
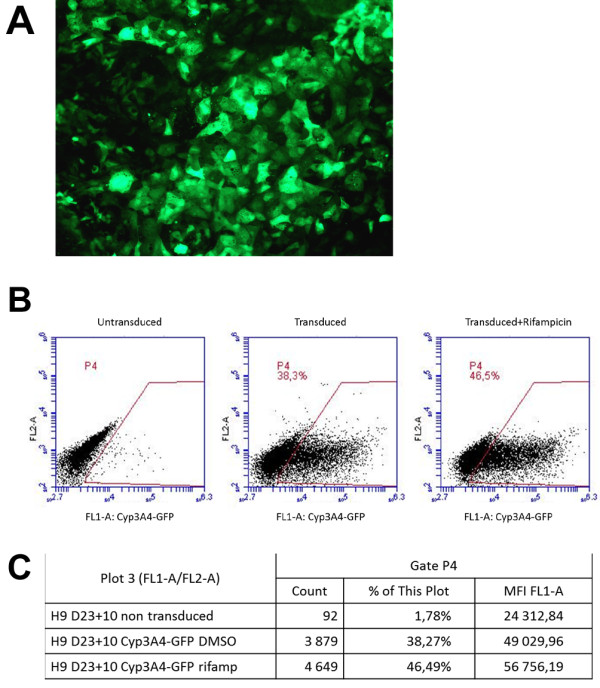
**Expression of green fluorescent protein (GFP) driven by the cytochrome P450 3A4 (CYP3A4) promoter in purified hepatocyte-like cells. (A)** Fluorescence microscopy of GFP-expressing cells on day 33 after transduction (day 25 of differentiation; day 9 after sorting) by a lentivector expressing GFP under the control of CYP3A4 promoter. **(B)** Fluorescence-activated cell sorting (FACS) analysis of GFP expression on day 33 of differentiation from non-transduced cells and from cells transduced on day 25 and treated ont day 30 for 72 hours with vehicle (DMSO) alone or with rifampicin. **(C)** Quantification of analysis in gate P4 **(B)** by the CFlow software (Accuri Flow Cytometer; BD Biosciences, Le Pont-de-Claix, France).

Finally, to further evaluate the functionality of differentiating hepatocytes, we sought to visualize expression of the mature hepatocyte-specific cytochrome P450 3A4 (CYP3A4). The cells were transduced on day 25 of differentiation (day 9 after sorting) with a lentivector expressing GFP under the control of the CYP3A4 promoter, and analyzed for fluorescence on day 33 (Figure 6A) [17]. Treatment of transduced cells with rifampicin, an inducer of CYP3A4, produced a modest increase in GFP-positive cells, both in proportion and in mean fluorescence intensity (MFI) suggesting that the CYP3A4 promoter is indeed regulated in these hepatocytes (Figure 6B, C).

## Discussion

Safe and efficient methods for the purification of progenitor cells are a prerequisite to allow use of differentiated hPSCs for regenerative medicine in order to minimize risks of tumorigenesis in patients. To date, strategies for purifying a given cell population have used either a cell surface protein specific for the target cell population, such as stage-specific embryonic antigen (SSEA)-1 for isolation of human multipotent cardiovascular progenitor cells [18], or lentivectors expressing a reporter gene under the control of a specific promoter [19]. In this study, we show for the first time that it is feasible to purify a population of hESC-derived hepatic progenitors that are devoid of viral integration and that can differentiate further into more mature hepatocyte-like cells. Our strategy was to engineer human hepatic progenitors generated from a hESC line to transiently express GFP under the control of liver-specific APOA-II. It was crucial to choose appropriate vectors that would exhibit high transduction efficiency at low MOI and would result in a vector-free enriched cell population. For this study, we developed a method for producing purified ILVs and IDLVs at high titers in order to minimize any deleterious effects upon transduction of target cells [20]. The *APOA-II* gene is expressed in liver and intestine, and we previously constructed a lentivector in which we inserted APOA-II regulatory sequences to drive GFP expression, and confirmed its functionality both *in vitro* and *in vivo*[12,13]. In the current study, to assess the specificity of our construct, various cell types were transduced, including human epithelial lines and different sources of mesoderm, such as primary human MSCs and fibroblasts. When driven by the APOA-II promoter, GFP was highly expressed only in hepatoma cells, confirming the suitability of this tool for purification of progenitors of the hepatic lineage and not for cells from mesodermal origin. Of note, GFP was not expressed in transduced human primary fibroblasts. Bi-potent mesendoderm, which can give rise to both definitive endoderm and mesoderm lineages, and transient populations expressing markers of both lineages, have been visualized *in vivo*[21,22].

The weak GFP expression seen in a low percentage of differentiating hES cells increased during the differentiation protocol, confirming the progressive differentiation of endoderm cells into hepatic cells. This was confirmed by the upregulation of HNF4α, a key hepatic transcription factor in hepatic progenitors [23]. Thus, our results showed that the cells generated in our culture system display the physiological regulation of a hepatic-specific promoter and also display markers of hepatoblasts. Such markers were first identified in developing mouse liver [24], and their expression in human progenitors has been confirmed by several groups, including us [25,26]. Our results also show that our purification approach does not prevent the sorted hepatoblasts from differentiating further into more mature hepatocytes, able to express FIX (a clotting factor specific to hepatocytes), export ICG, secrete albumin, and express and regulate the CYP3A4 promoter. The hepatocytes we generated were not as fully mature as adult hepatocytes, but to our knowledge, this stage has not yet been achieved with pluripotent stem cell-derived hepatocytes [27].

HIV integrase catalyses the enzymatic reactions that result in the covalent integration of the viral DNA into the host DNA [28]. HIV-1 reporter viruses harboring mutations of integrase active-site residues are unable to catalysz viral DNA integration, but still yield a reproducible level of reporter gene expression from the non-integrated proviral forms via DNA episomal forms. These viral episomes are progressively lost in dividing cells, leaving only the background level associated with non-specific random integration. Two types of circular episomes with intact coding regions are also produced. Homologous recombination within the LTRs generates a circular episome with a single LTR (1-LTR circle). Non-homologous end joining of the linear episome results in a circular episome with two adjacent LTRs.

Our results show that, 17 days after progenitor transduction, lentivector integration was undetectable. These results are in agreement with those of Mátrai *et al.*, who infused IDLVs into liver and provided molecular evidence that the background integration was not mediated by residual catalytic activity of the mutant integrase [29]. Inhibition of integration by specific integrase inhibitors irreversibly blocks HIV-1 replication. We reasoned that, as an alternative to IDLV, use of an HIV integrase inhibitor in combination with ILV should also prevent any integration events. Raltegravir, which specifically targets the strand transfer reaction, and is currently being used in clinical trials [30], abolished vector integration in our ILV-transduced progenitors, showing that use of ILV in combination with HIV integrase inhibitor can be an alternative to IDLV. Thus, our methods can be used to purify hepatic progenitors prior to their differentiation into hepatocytes, and offers the advantage that resulting cells are free of lentivector DNA integration. Given that xenogenic reagents such as mouse embryonic fibroblasts (MEFs) or Matrigel (BD Biosciences) are incompatible with the use of hPSCs to treat debilitating human diseases [31], our system, combined with the establishment of synthetic matrices, should facilitate the development of protocols for the generation of mature hepatocytes for future clinical applications.

## Conclusions

Our approach, based on purified IDLV, facilitates the generation of a population of purified hESC-derived hepatic progenitors, which are devoid of integrated viral DNA, and can be subsequently differentiated to mature hepatocyte-like cells. This method represents a technological advance for many areas of research because it can be adapted easily for the purification of stem cell-derived populations such as pancreatic or neuronal cells for therapeutic purposes and also for *in vitro* applications such as drug screening.

## Methods

### Cell culture

Human H9 embryonic stem cells (WA09, WiCell, Madison, WI, USA) were cultured on MEFs treated with mitomycin C. Cells were manually dissected and plated onto 0.1% gelatin-coated culture dishes (Costar, Corning Life Sciences, Acton, MA, USA) for amplification. The human ES cell culture medium was composed of standard Dulbecco’s modified Eagle’s medium (DMEM)/F12 supplemented with 20% Knockout Serum Replacement (Gibco/Life Technologies Corp., Saint Aubain 91190, France), 4 ng/ml human recombinant basic fibroblast growth factor (bFGF; R&D Systems Inc., Minneapolis, MN, USA), 0.1 mmol/l β-mercaptoethanol, 1 mmol/l L-glutamine, and 1% non-essential amino acids. Fetal livers were obtained from pregnancies terminated at 11 to 13 weeks of gestation, after obtaining the informed consent of the mothers, as recommended by the French Ethics Committee and the local Ethics Committee of Paris XI University (Paris, France). Human hepatoblasts were then isolated and cultured as previously described [25,32]. Primary human adult hepatocytes were isolated from normal liver tissue biopsy specimens obtained during resections (partial hepatectomies) for hepatic or metastatic tumors, after informed consent had been obtained from the patient. MSCs, HeLa cells, human cervical epithelial tumor cells, HuH7cells (a human hepatoma cell line), and COP cells (a cell line generated from SV40 transformation of human pancreatic islets) were used to seed a six-well microplate (CellBind; Corning Life Sciences) containing DMEM (PAA Laboratories, Les Mureaux, France) supplemented with 10% fetal bovine serum (FBS). They were cultured at 37°C for 24 hours, under an atmosphere containing 5% CO_2_. Transduction was performed overnight at an MOI of 40 for MSCs and HeLa cells, and at an MOI of 30 for HuH7 cells, in the presence of 8 μg/ml hexadimethrine bromide (Polybrene; Sigma-Aldrich Chimie, Lyon, France). The supernatant was then replaced with fresh culture medium.

A549 cells (a human alveolar epithelial cell line) were used to seed six-well microplates (CellBind; Corning Life Sciences) containing HamF12 (PAA Laboratories) supplemented with 10% FBS, and then cultured at 37°C for 24 hours, under an atmosphere containing 5% CO_2_. Cells were transduced overnight at an MOI of 40, in the presence of 8 μg/ml hexadimethrine bromide (Polybrene; Sigma-Aldrich). The supernatant was then replaced with fresh culture medium.

MCF7 cells (a breast cancer epithelial cell line) were used to seed six-well microplates (CellBind; Corning Life Sciences) containing MEM (PAA Laboratories) supplemented with 10% FBS + 0.01 mg/ml bovine insulin (Sigma-Aldrich) and were cultured at 37°C for 24 hours, under an atmosphere containing 5% CO_2_. Cells were transduced overnight at an MOI of 80, in the presence of 8 μg/ml hexadimethrine bromide (Polybrene; Sigma-Aldrich). The supernatant was then replaced with fresh culture medium. At 1 week after transduction, GFP expression was analyzed by flow cytometry or fluorescence microscopy. Phase-contrast images were taken under a microscope (Eclipse; Nikon, Tokyo, Japan).

Human fibroblasts were cultured in DMEM medium supplemented with 10% FBS and antibiotics. HUVECs were cultured in chemically defined EBM2 endothelial basal medium with antibiotics. Cells were transduced overnight at an MOI of 30. At 4 days after transduction, GFP expression was analyzed by flow cytometry. COP cells were cultured in low-glucose DMEM supplemented with 10% FBS and antibiotics.

### Differentiation of hESCs into hepatic progenitor cells

At 1 day before the passage of hESCs for differentiation, 30 × 60 mm cell culture dishes (CLS430166-500EA; Corning Life Sciences) were coated with 0.1% gelatin from porcine skin type A (61890-100G; Sigma-Aldrich). After 90 min of incubation at room temperature, the gelatin was removed, and dishes were washed once with phosphate-buffered saline (PBS). A coating medium (DMEM, 10% FBS, 100 × MEM non-essential amino acid solution, 1% L-glutamine) was added to plates, which were then incubated for 24 hours at 37°C, under an atmosphere containing 5% CO_2_. The following day, hESCs were dissected from MEFs. For this, the hESC culture medium was removed from the cells and replaced with chemically defined medium (CDM) supplemented with bovine serum albumin (BSA; Europabioproducts, Ely, UK; 5 mg/ml final concentration), fibroblast growth factor (FGF)2 (12 ng/ml final concentration) and activin A (10 ng/ml final concentration). About 80 colonies of hESCs on MEFs per 60 mm dish were dissected with a sterile pipette tip. The coating medium was removed from the gelatin-coated dishes, which were washed once with 1 × PBS, then CDM-BSA containing the previously dissected hESC clusters was added to the plates. We used 40 dissected colonies per pre-coated plate. After incubation for 48 hours, the CDM-BSA was removed and replaced with CDM supplemented with polyvinylalcohol (PVA; Sigma-Aldrich; 1 mg/ml final concentration: CDM-PVA), activin A (100 ng/ml final concentration), FGF2 (20 ng/ml final concentration), bone morphogenetic protein (BMP)4 (10 ng/ml final concentration), and LY294002 (10 μmol/l final concentration). The medium was replaced daily. After 3 days, cells were incubated with CDM-PVA supplemented with FGF10 (50 ng/ml) for a further 3 days. Retinoic acid (0.1 μmol/l final concentration) and SB431542 (10 μmol/l final concentration) were then added, together with FGF10, and the cells incubated for an additional 2 days. Finally, cells were incubated for 4 days with CDM-PVA supplemented with hydrocortisone (1 μmol/l final concentration), FGF4 (30 ng/ml final concentration), HGF (50 ng/ml final concentration) and epidermal growth factor (EGF; 50 ng/ml final concentration).

On day 16 of differentiation, attached cells were removed in a cell dissociation buffer (0.1 mg/ml EDTA and 0.5 mg/ml BSA in PBS), and GFP-expressing cells were purified with a cell sorter (FACS DiVa Flow Cytometer; Becton Dickinson, Franklin Lakes, NJ, USA). Purified hepatic progenitors in a plating medium (DMEM/HAM F-12, 10% heat-inactivated FBS, 1 g/l human BSA fraction V, 1% L-glutamine) were plated onto a type I collagen-coated plate and incubated for 4 hours. Cells were then incubated overnight with hepatic progenitor medium (HPM: DMEM/HAM F-12/Williams Medium E 1:1, 0.24% linoleic acid-albumin 2:1, 5.10^-8^ mol/l triiodo-L-thyronine, 0.2 IU insulin, 10^-6^ mol/l hydrocortisone, 6 × 10^-4^ mol/l vitamin C, 6 × 10^-4^ mol/l human apo-transferrin) supplemented with HGF (50 ng/ml). For the next 2 days, cells were incubated with HPM supplemented with HGF (50 ng/ml) and EGF (20 ng/ml). Cells were then incubated with hepatocyte culture medium (Lonza Group Ltd, Basel, Switzerland) supplemented with the associated kit (HCM Bullet Kit; Lonza) and Oncostatin M (10 ng/ml). Phase-contrast images were taken under a microscope (Eclipse; Nikon).

### Lentivector production

The APOA-II promoter was previously cloned in lentivectors by our laboratory [12]. The EF1α-GFP and APOA-II-GFP cassettes were inserted into third-generation self-inactivating (SIN) lentivectors containing a WPRE sequence and a mutated GAG sequence. These vectors were designed and produced by Vectalys SAS (Toulouse, France).

Viral vectors were produced in a human embryonic kidney (HEK)293T cell line. The HEK293T cells were used to seed a 10-layer cell culture chamber (6320 cm^2^; CellSTACK; Corning Life Sciences) and were transfected 2 days later, in fresh DMEM without fetal calf serum (FCS) supplemented with 1% penicillin/streptomycin and 1% ultraglutamine (PAA Laboratories). Cells were simultaneously transfected with three plasmids: pVSVG, pGagPol, and pLV-APOA-II-GFP. The supernatant was discarded 24 hours after transfection, and replaced with fresh non-supplemented DMEM. The harvested vectors were clarified by centrifugation for 5 minutes at 3000 *g*, followed by microfiltration through a sterile filter unit with 0.45 μm pores (Stericup; Millipore Corp., Billerica, MA, USA). The crude vector preparation was concentrated and purified by tangential flow ultrafiltration, and the supernatant was then diafiltered against DMEM. Once the diafiltration was complete, the retentate was recovered, and further concentrated by ultrafiltration.

### Quantification of functional particle by FACS

HCT116 cells were used to seed 96-well plates at a density of 12,500 cells per well, in 250 μl of DMEM supplemented with 10% FCS, 1% penicillin/streptomycin, and 1% ultraglutamine (complete medium). Five serial dilutions with complete medium were performed 24 hours later for each vector sample and an rLV-EF1-GFP internal standard. The cells were transduced with these serial dilutions in the presence of 8 μg/ml hexadimethrine bromide (Polybrene; Sigma-Aldrich). For each sample series, one well of non-transduced cells was included as a control. At 4 days after transduction, the cells were released by trypsin treatment and harvested by centrifugation, then each cell pellet was resuspended in 250 μl of PBS. The titer was calculated by determining the number of transducing units (TU)/ml by FACS.

### Quantification of physical particles by p24 ELISA

The p24 core antigen was detected directly in the viral supernatant with a HIV-1 p24 ELISA kit (Perkin Elmer, Waltham, MA, USA) in accordance with the manufacturer’s instructions. The absorbance of each microplate well was determined with a microplate reader, and calibrated against tan HIV-1 p24 antigen standard curve. The viral titer, expressed in physical particles per ml, was calculated from the amount of p24, assuming that 1 pg of p24 corresponds to 10^4^ physical particles.

### Transduction of hESCs by lentivectors

Before transduction, hESCs were manually dissociated and incubated, in clumps, with viral particles for 2 hours at 37°C in low-attachment 24-well plates (Corning Life Sciences), with gentle rocking. They were then added to MEFs in hESC medium. The undifferentiated transduced cell population was expanded and differentiated in CDM [12] devoid of serum and supplemented with insulin, transferrin, and defined lipids, to which was added BSA (5 mg/ml fraction V; Europabioproducts) for expansion or PVA (1 mg/ml, mean molecular weight 30,000 to 70;000; P8136-250G, Sigma) as a substitute for BSA.

### Transduction of human ESC-derived hepatic progenitor cells by IDLV

On day 13 of differentiation, cells were washed once with PBS, and fresh CDM-PVA supplemented with HGF (50 ng/ml final concentration), EGF (50 ng/ml final concentration), FGF4 (30 ng/ml final concentration), and hydrocortisone (10^-6^ mol/l final concentration) were added. The IDLV was used at an MOI of 30 and was incubated with cells for 24 hours. The cells were cultured for a further 2 further days, with the medium changed daily. The HIV integrase inhibitor raltegravir was added to the culture medium on the day of transduction, at a concentration of 1 μmol/l, and was maintained in the medium for 24 hours.

### Cell preparation for sorting and plating

Differentiated cells were washed with PBS and incubated for 5 min at 37°C with 2 ml per 60 mm dish of cell dissociation buffer (0.01 mg/ml EDTA, 0.05 mg/ml BSA in PBS without Ca^2+^/Mg^2+^, pH 7.5). Dissociated cells were suspended in 5 ml of plating medium (DMEM/F12; 20% human serum, PAA Laboratories), and separated by centrifugation for 5 minutes at 400 *g*. The cell pellet was finally suspended in plating medium (DMEM/F12, 20% human serum 1 mmol/l L-glutamine, 100 μl/ml antibiotics) supplemented with HGF (50 ng/ml final concentration), at a density of 3 × 10^6^ cells/ml for FACS. GFP-positive cells were plated onto a 24-well type I collagen-coated plate (BD Biosciences) at a density of 2 × 10^5^ cells per well. At 4 hours after plating, the medium was replaced with CDM-PVA supplemented with HGF (50 ng/ml final concentration), EGF (50 ng/ml final concentration), and FGF4 (30 ng/ml final concentration), and cells were cultured for a further 2 days before analysis. Phase-contrast image were taken under a microscope (Eclipse; Nikon).

### Southern blotting

Southern blot hybridization was carried out using digoxigenin (DIG System; Roche Applied Science, Basel, Switzerland) in accordance with the manufacturer’s protocol. Briefly, 5 μg of genomic DNA from each sample was digested with either *Eco*RI (New England Biolabs, Beverly, MA, USA) for the detection of 1- and 2-LTR circular DNA (4,396 bp and 4,627 bp bands, respectively) or with *Eco*RI and *Bam*HI (New England Biolabs) for the detection of total lentiviral DNA as a 1,403 bp band. The digested DNA was subjected to electrophoresis in a 1% agarose gel. Gels were blotted onto Hybond-N + membrane (Millipore) overnight in 20 × SSC buffer (Sigma-Aldrich). As a probe, we used 1.3 kb PCR (GFP-WPRE) fragments (see Additional file 1: Figure S1B) labeled with DIG-11-dUTP (PCR DIG Probe Synthesis Kit; Roche). The probe was hybridized with the membrane overnight at 42°C (DIG Easy Hyb solution; Roche), and the probe detected ()DIG Detection System; Roche). The DIG-labeled probe was detected with an anti-digoxigenin-AP Fab fragment (Roche) and visualized with achemiluminescent substrate (CSPD; Roche).

### Evaluation of copy number

The copy number of the lentiviral vector was determined by qPCR analysis. The qPCR experiments were performed with 150 ng of total DNA, SYBR GreenER (Invitrogen Corp., Carlsbad, CA, USA), specific primers binding to the WPRE sequence, and albumin (housekeeping gene) in a final volume of 20 μl (Step One Real-Time PCR System; Applied Biosystems, Foster City, CA, USA). Copy number was calculated by referring the Ct values for each sample to a standard plasmid curve. All qPCRs were performed in duplicate.

To determine the limits of detection of residual lentivector integration, serial dilutions were performed using genomic DNA from a clonal cell line containing only one copy of ILV. The line was established by transduction of HCT116 cells with a GFP-expressing ILV, followed by clonal selection, and the copy number was quantified by Southern blotting. Serial dilutions of genomic DNA bearing one copy per cell were performed by two-fold dilutions in genomic DNA from control non-transduced HCT116 cells. A range of dilutions from 1:2 to 1:10000 were tested. All qPCRs were performed in duplicate.

### RT-PCR

Total RNA was extracted (RNeasy Mini Kit (Qiagen Inc., Valencia, CA, USA) from hESCs, differentiated hepatic progenitors, and human fetal and adult hepatocytes, following the manufacturer’s protocol. For each sample, 0.6 μg of total RNA was reverse-transcribed with reverse transcriptase (Superscript II; Invitrogen), and amplification by PCR was performed (PCR Nucleotide Mix Kit; Promega, Charbonnières, France). The primers used and the sizes of the amplicons obtained are described in Table 1.

**Table 1:** Primer sequences used for reverse transcription-PCR in this study

**Gene**	**Direction**	**Primer sequence 5′→3′**	**Annealing temperature, °C**	**Amplicon size, bp**
*FoxM1B*	Forward	GGGCGCACGGCGGAAGATGAA	55	492
	Reverse	CCACTCTTCCAAGGGAGGGCTC		
*Fibrinogen β*	Forward	CAAGGTGTCAACGACAATGAGGAG	55	315
	Reverse	CAGGTCTGGGTCAGCGTGAAGAGA		
*HNF4α*	Forward	CTGCTCGGAGCCACCAAGAGATCCATG	55	370
	Reverse	ATCATCTGCCACGTGATGCTCTGCA		
*AFP*	Forward	AGAACCTGTCACAAGCTGTG	55	675
	Reverse	GACAGCAAGCTGAGGATGTC		
*ApoA-II*	Forward	GGAGAAGGTCAAGAGCCCGAG	55	246
	Reverse	AGCAAAGAGTGGGTAGGGACAG		
*FIX*	Forward	GTCCTGTGAACCAGCAGTGCC	56	512
	Reverse	TTGTCAGCAATGCAAATAGGT		
*Β-actin*	Forward	TCACCACCACGGCCGAGCG	58	350
	Reverse	TCTCCTTCTGCATCCTGTCG		

### PCR and real-time qPCR

Real-time PCR mixtures were prepared (SensiMiX Kit; Bioline, Paris, France), in accordance with the manufacturer’s instructions. Primers used for real-time PCR analyses are shown in Table 2. The DNA was then denatured at 95°C for 10 minutes, and subjected to 40 cycles of 95°C for 30 seconds, 60°C for 30 seconds, and 72°C for 30 seconds, followed by a final extension at 72°C for 10 minutes. Real-time PCR was performed in a real-time PCR system (model 7300 Applied Biosystems), in triplicate, with normalization to hypoxanthine-guanine phosphoribosyltransferase (hPRT) levels in the same run. The real-time qPCR results are presented as the means of three independent experiments; error bars indicate the SEM.

**Table 2:** Quantitative PCR primer sequences used in this study

**Gene**	**Direction**	**Primer sequence 5′→3′**
*CK19*	Forward	TGAGTGACATGCGAAGCCAATAT
	Reverse	GCGACCTCCCGGTTCAAT
*HPRT*	Forward	TAATGGTGGAGATGATCTCTCAAC
	Reverse	TGCCTGACCAAGGAAAGC

### FACS analysis of sorted cells

After transduction with the IDLV ApoA-II-GFP lentivector, the differentiating hepatocytes were analyzed for GFP expression 14 days after progenitor sorting, using a cytometer (n Accuri C6; Becton Dickinson) and associated software (CFlowPlus; Becton Dickinson).

### Transduction of purified progenitors

Purified progenitors were transduced 5 days after sorting, with the Cyp3A4-GFP lentivector (kindly provided by Dr Anne Corlu, INSERM U 991), and fluorescence was assessed 12 days later. Rifampicin (10 μmol/l) or DMSO was added to the plates at day 30 for 48 hours, and cells were analyzed 1 day later (day 33).

### Immunocytochemistry

Cells were fixed by incubation with 4% paraformaldehyde for 10 minutes at room temperature, then washed with 50 mmol/l NH_4_Cl in PBS for 10 minutes, and permeabilized by incubation with 0.1% Triton X-100 for 4 minutes. Cells were blocked by incubation with 3% serum in PBS for 1 hour. Primary antibodies were diluted in PBS supplemented with 4% BSA (see Table 3 for the corresponding dilution) and incubated with cells for 2 hours at room temperature. Cells were then washed with PBS and incubated with secondary antibodies diluted in PBS supplemented with 4% BSA (see Table 3 for corresponding dilution) for 1 hour at room temperature. Cell nuclei were stained with 4',6-diamidino-2-phenylindole (DAPI), and cells were mounted in (Vectashield Mounting Medium; Vector Laboratories/AbCys, Paris, France). Fluorescence micrographs were obtained with AxioVision Rel. 4.8 microscopy software.

**Table 3:** Antibodies used in this study

**Antibody**	**Company**	**Reference number**	**Dilution**
Primary			
EpCam	AbCys	abc171	1:20
CK19	Dako^a^	M0888	1:50
AFP	Dako	N1501	1:300
HNF4α	Santa Cruz^b^	SC-8987	1:100
E-cadherin	Dako	M3612	1:100
SOX17	R&D	MAB1924	1:100
OCT4	Santa Cruz	SC-5279	1:200
NANOG	R&D	AF1997	1:100
SSEA 4	AbCys	VMA 4304	1:100
TRA 1-60	Millipore	MAB4360	1:100
Secondary			
Goat anti-mouse	Molecular Probes^c^	A21123	1:800
Chicken anti-goat	Invitrogen	A21467	1:800
Goat anti-rabbit	Molecular Probes	A11010	1:800

^a^Dako, Glostrup, Denmark.

^b^Santa Cruz Biotechnology Inc., Santa Cruz, CA, USA.

^c^Molecular Probes, Eugene, OR, USA.

### ICG uptake and release

The ICG uptake test was performed on cells 17 days after sorting, by incubating the cells with 1 mg/ml ICG for 60 min at 37°C. Cells were then washed in medium, and ICG release was evaluated 16 hours later.

### Albumin secretion

Albumin concentrations in cell culture medium were measured with a kit specific for human albumin detection, in accordance with the manufacturer’s instructions (Dade Behring SAS, France) in the Department of Biochemistry, Bicêtre Hospital.

Glycogen storage was assayed by the periodic acid-Schiff technique according to McManus [33].

## Competing interests

The authors declare that they no competing interests with respect to this manuscript.

## Authors’ contributions

GY and KST were involved in conception and design, data collection, analysis and interpretation, and manuscript writing; SC, RV, RG, ND, CM, DC, and SM in data collection and data analysis; GT in data analysis and interpretation; DB in data analysis and interpretation, and manuscript writing; LV in provision of study material; PB in provision of study material and data analysis and interpretation; and AW and ADK in conception and design, data analysis and interpretation, and manuscript writing. All authors read and approved the final manuscript.

## Supplementary Material

Additional file 1: Figure S1 Transient green fluorescent protein (GFP) expression after transduction of human embryonic stem cells (hESCs) with elongation factor (EF)1α-GFP integrase-defective lentivectors (IDLVs). **(A)** Time course of fluorescence-activated cell sorting (FACS) analysis used to analyze fractions of fluorescent GFP-expressing cells after transduction of H9 cells with EF1α-GFP-IDLV at a multiplicity of infection (MOI) of 10 and 30. **(B)** Time course of FACS analysis showing similar proportions of GFP-IDLV cells and GFP-integrating lentivectors (ILV) cells 3 days after transduction, and a decrease in the proportion of GFP-IDLV cells 10 and 14 days after transduction, and of EF1α-GFP-ILV cells in the presence of raltegravir. Click here for file

Additional file 2: Figure S2 **(A)** Genetic map of 1-long terminal repeat (LTR) and 2-LTR circular DNA. **(B)** Map of the probe used for the Southern blotting experiments. After double *Bam*HI/*Eco*RI digestion, a 1,316 bp band common to integrated and non-integrated forms of the lentivector DNA was detected. After single *Eco*RI digestion, only non-integrated forms (1-LTR and 2-LTR circle) of the lentivector DNA were detected. Click here for file

Additional file 3: Figure S3 Limits of detection of virus copy number. Serial dilutions (1:50 to 1:10000) of genomic DNA from a clonal cell line containing one copy number of an integrating green fluorescent protein (GFP)-expressing lentivirus (D2) and derived from HCT 116 cekks. HCT 116 NT: control non-transduced cells. Click here for file
