# Supplementary figures and images for: Integration-deficient lentivectors: an effective strategy to purify and differentiate human embryonic stem cell-derived hepatic progenitors
Source: BMC Biol. 2013 Jul 19;11:86. doi: 10.1186/1741-7007-11-86 (PMC3751548; doi:10.1186/1741-7007-11-86)

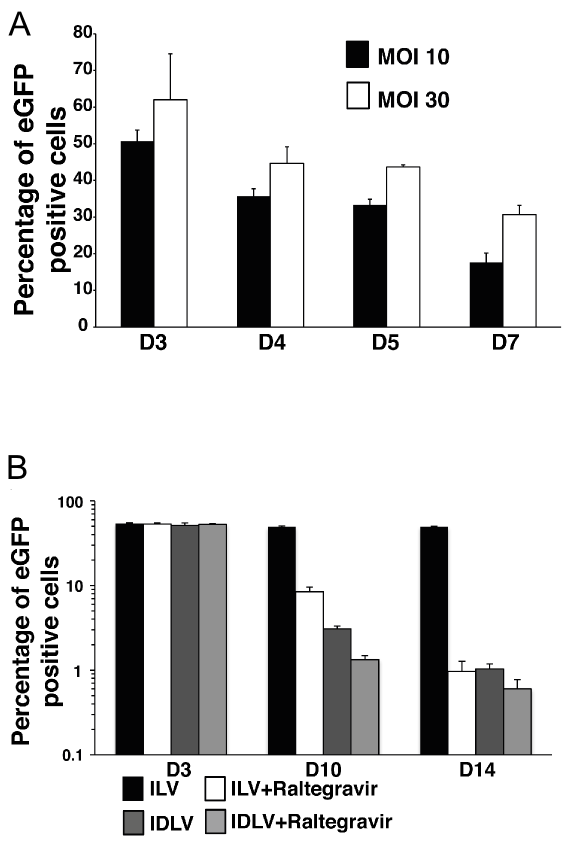

Supplement: Additional file 1: Figure S1 — Transient green fluorescent protein (GFP) expression after transduction of human embryonic stem cells (hESCs) with elongation factor (EF)1α-GFP integrase-defective lentivectors (IDLVs). (A) Time course of fluorescence-activated cell sorting (FACS) analysis used to analyze fractions of fluorescent GFP-expressing cells after transduction of H9 cells with EF1α-GFP-IDLV at a multiplicity of infection (MOI) of 10 and 30. (B) Time course of FACS analysis showing similar proportions of GFP-IDLV cells and GFP-integrating lentivectors (ILV) cells 3 days after transduction, and a decrease in the proportion of GFP-IDLV cells 10 and 14 days after transduction, and of EF1α-GFP-ILV cells in the presence of raltegravir. [file 1741-7007-11-86-S1.tiff]

**A**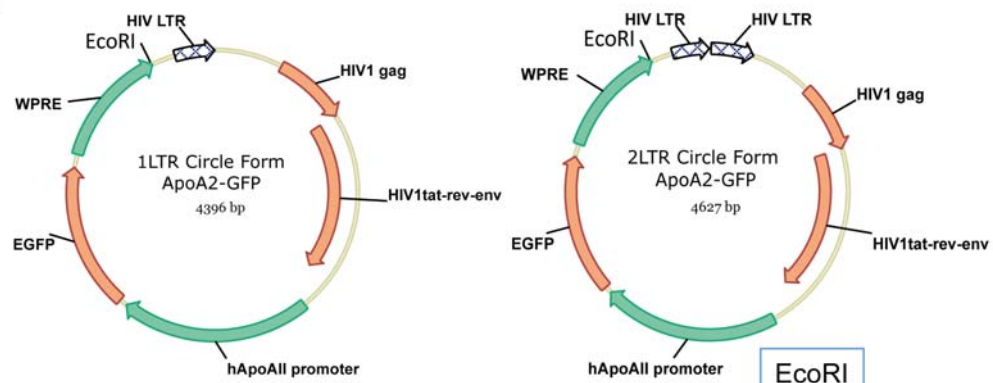**B**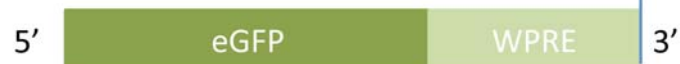

Supplement: Additional file 2: Figure S2 — (A) Genetic map of 1-long terminal repeat (LTR) and 2-LTR circular DNA. (B) Map of the probe used for the Southern blotting experiments. After double BamHI/EcoRI digestion, a 1,316 bp band common to integrated and non-integrated forms of the lentivector DNA was detected. After single EcoRI digestion, only non-integrated forms (1-LTR and 2-LTR circle) of the lentivector DNA were detected. [file 1741-7007-11-86-S2.pdf]

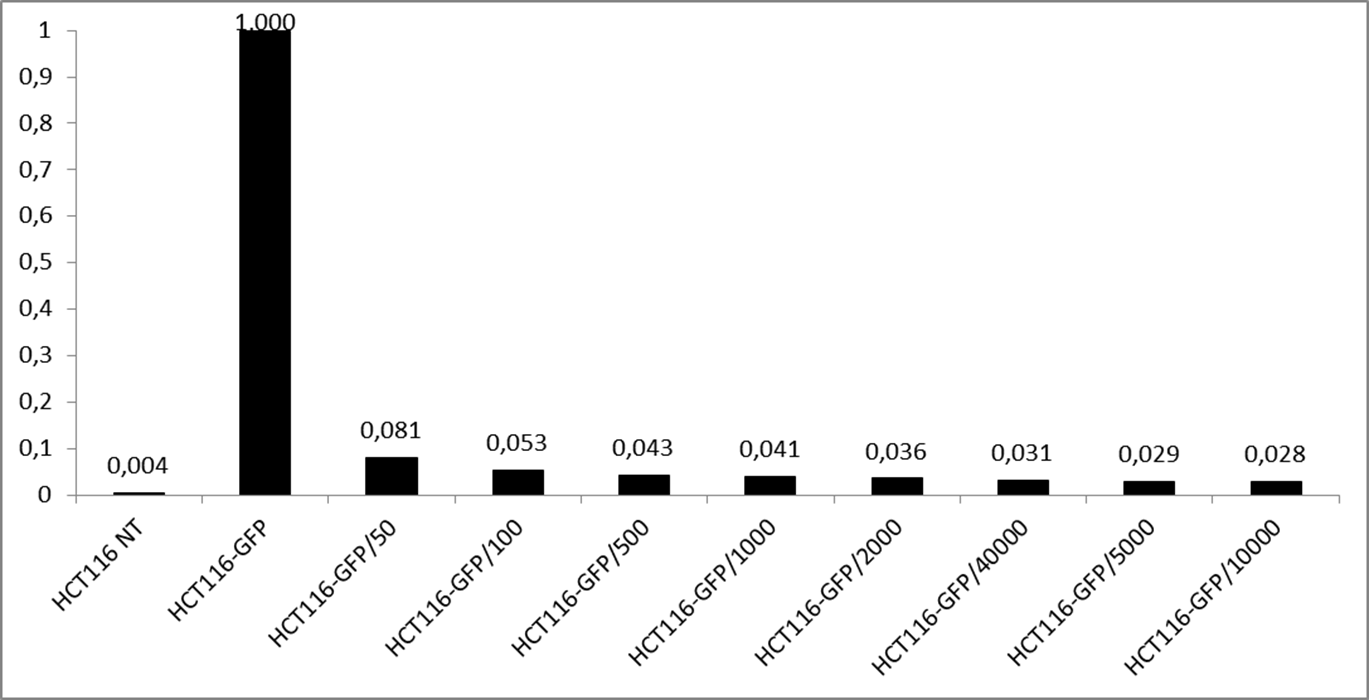

Supplement: Additional file 3: Figure S3 — Limits of detection of virus copy number. Serial dilutions (1:50 to 1:10000) of genomic DNA from a clonal cell line containing one copy number of an integrating green fluorescent protein (GFP)-expressing lentivirus (D2) and derived from HCT 116 cekks. HCT 116 NT: control non-transduced cells. [file 1741-7007-11-86-S3.tiff]
